# Multi-Channel Nuclear Analysis: An ImageJ/FIJI Plugin for Automated Nuclear Segmentation and Multi-Channel Fluorescence Analysis

**DOI:** 10.17912/micropub.biology.001590

**Published:** 2025-06-06

**Authors:** Ariel Waisman

**Affiliations:** 1 Laboratory of Applied Neuroscience Research (LIAN), Foundation for the Fight Against Childhood Neurological Diseases (FLENI), Institute of Neurosciences (INEU), CONICET, Buenos Aires, Argentina.

## Abstract

Quantitative analysis of fluorescence microscopy images is essential for studying expression levels, subcellular localization and co-occurrence of proteins and other biomolecules. While several automated tools exist for specific applications, there remains a need for user-friendly, customizable tools that can analyze multi-channel fluorescence images with nuclear segmentation capabilities. Here we present Multi-Channel Nuclear Analysis, an open-source ImageJ/FIJI plugin that combines the robust nuclear segmentation capabilities of StarDist with versatile multi-channel analysis features. The tool provides a graphical user interface for configuring analysis parameters, processes multiple images in batch mode, and generates both individual and consolidated measurement tables to facilitate downstream analysis. A companion tool for merging separate channel files into multi-channel images extends compatibility to diverse microscopy systems. Together, these tools enable researchers without extensive programming experience to perform comprehensive quantitative analysis of nuclear-centered multi-channel fluorescence images.

**Figure 1. Multi-Channel Nuclear Analysis workflow and case study:**
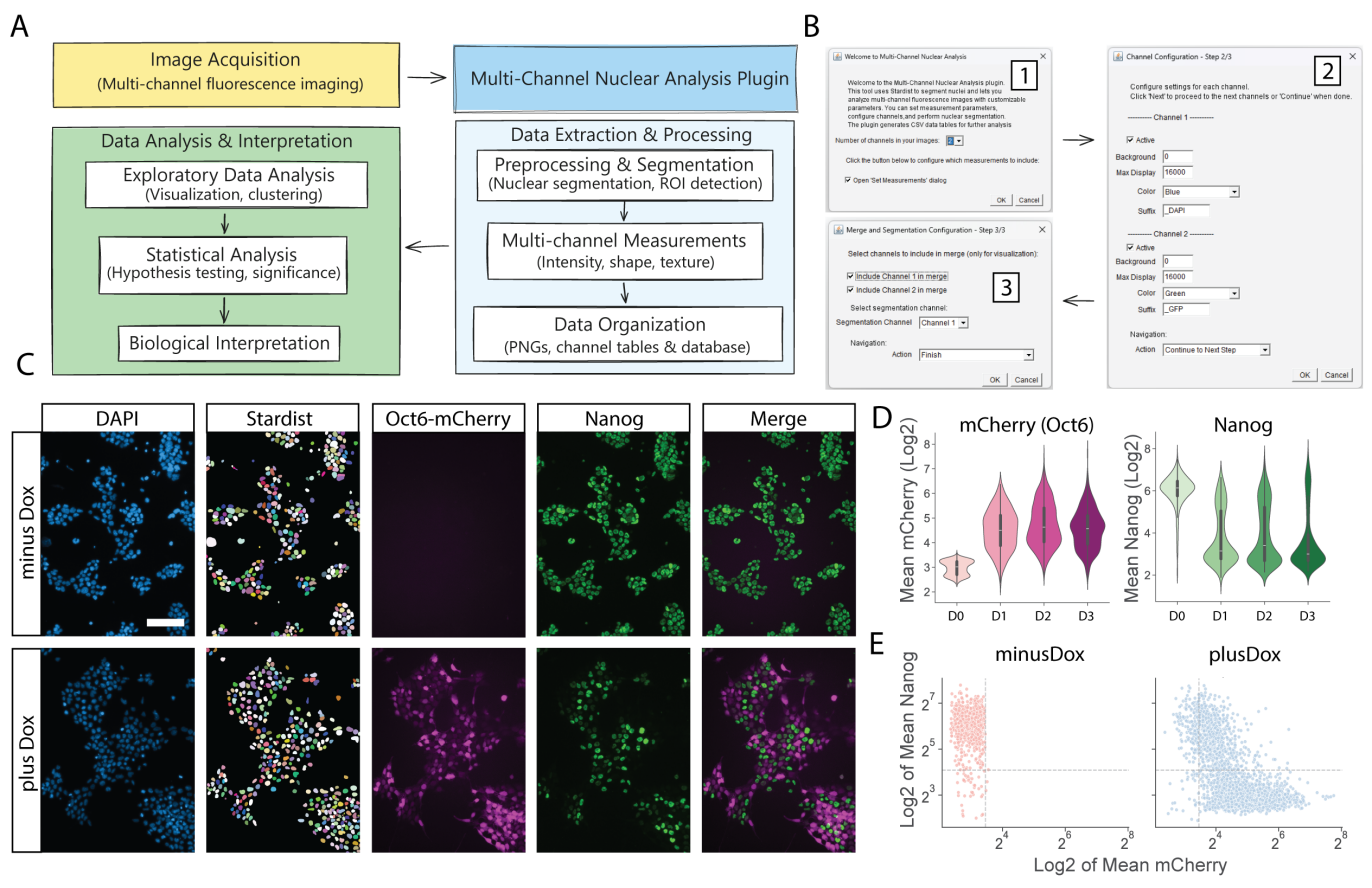
(A) Schematic representation of the analysis workflow. (B) Configuration dialog boxes for parameter customization. (C) Representative output images showing individual channels, segmentation results, and merged visualization. Scale bar: 100 µm (D) Violinplots created with Python and Seaborn for the expression of OCT6-mCherry and NANOG across different Dox treatment durations. (E) Scatterplot demonstrating the inverse correlation between NANOG and OCT6-mCherry expression in individual cells.

## Description

Fluorescence microscopy is a central technique in cell biology for visualizing and quantifying subcellular structures and molecular interactions. Multi-channel fluorescence imaging allows researchers to simultaneously observe the distribution of multiple biomolecules and assess their colocalization or relative abundance. Quantitative analysis of fluorescence microscopy images requires multiple processing steps, from image acquisition to data extraction, visualization and statistical analysis ( Figure 1A ).

Despite advancements in image analysis software, many researchers still face challenges in efficiently extracting quantitative data from multi-channel images, particularly when nuclear segmentation is required. The interdisciplinary gap between software developers and life scientists often results in tools that fail to meet the specific needs of biological research (Pylvänäinen et al., 2025).

While specialized software solutions exist for image analysis, they are destined for different user communities. Expert image analysts and computational biologists typically rely on Python-based frameworks for their flexibility and extensibility. However, experimental biologists and microscopy core facility users more commonly utilize ImageJ/FIJI (Schindelin et al., 2012; Schneider et al., 2012), which offers several key advantages: a graphical user interface, cross-platform compatibility, extensive plugin ecosystem, and a lower barrier to entry for those without programming expertise. ImageJ's 38-year history has established it as a cornerstone in biological image analysis (Schneider et al., 2012), while the FIJI distribution ("FIJI Is Just ImageJ") extends its capabilities with pre-installed plugins and updates that make it particularly valuable for fluorescence microscopy applications (Schindelin et al., 2012).

Several factors contribute to the challenge of effective multi-channel analysis: (1) the need for accurate nuclear segmentation, (2) the requirement to measure multiple parameters across different channels, and (3) the necessity to organize measurements in a format suitable for exploratory and statistical analysis. While specialized software packages exist for these tasks, they often require programming expertise, are platform-specific, or lack user-friendly interfaces for parameter customization.

To address these needs, we developed Multi-Channel Nuclear Analysis, an ImageJ/FIJI plugin that leverages the robust nuclear segmentation capabilities of StarDist (Schmidt et al., 2018) combined with customizable multi-channel analysis features. The tool was developed following best practices for scientific software development (Pylvänäinen et al., 2025), with a focus on user-centric design and accessibility. It provides a graphical user interface (GUI) for parameter configuration, processes multiple images in batch mode, and generates both individual and consolidated measurement files to facilitate various analytical approaches. By building upon the established ImageJ/FIJI ecosystem, our tool bridges the gap between advanced image analysis capabilities and the practical needs of bench scientists working with fluorescence microscopy data.

To demonstrate the utility of the Multi-Channel Nuclear Analysis tool, we applied it to multi-channel fluorescence images from a previous study investigating transcription factor dynamics in mouse embryonic stem cells (mESCs) (Waisman et al., 2024). In this experimental system, mESCs were engineered to express the OCT6 transcription factor ( TF ) fused with mCherry upon doxycycline (Dox) induction. When this TF was expressed in naive pluripotency culture conditions, we observed a significant downregulation of the pluripotency marker NANOG . We analyzed cells under four conditions: untreated (minus Dox) and treated with Dox for 24, 48, or 72 hours. The cells were subjected to immunofluorescence staining against NANOG , while also imaging for OCT6-mCherry, with DAPI counterstaining to visualize nuclei.

Figure 1B shows the main configuration dialogs that guide users through the setup process. The intuitive interface allows users to specify the number of channels, background subtraction values, and display settings, facilitating reproducible analysis across multiple images.

The tool successfully segmented nuclei using StarDist and extracted measurements from all channels. Figure 1C shows representative output images, including individual channels, segmentation results, and merged visualization. The resulting measurements are organized in CSV tables with a clear structure that facilitates further analysis.

To illustrate the analytical possibilities, we generated visualizations using Python and the Seaborn library based on the consolidated data table (Complete_data.csv). Importantly, the results that are output in the CSV tables can also be analyzed with more user-friendly software, such as Microsoft Excel or Graphpad Prism. The resulting visualizations revealed that OCT6-mCherry was rapidly induced following Dox treatment and led to NANOG repression as early as 24 hours post-treatment ( Figure 1D ). Furthermore, a scatterplot of mCherry and NANOG expression in individual cells demonstrated that NANOG downregulation was strongly correlated with OCT6-mCherry expression levels, suggesting a direct relationship between these factors.

The complete workflow, from image import to data visualization, can be completed in under 30 minutes and requires minimal user intervention after the initial configuration. The plugin processing speed depends on the number and size of images but typically takes only a few minutes for a standard dataset, making it suitable for high-throughput analysis. For instance, for the set of images used this study, which are also available as “Example Image Dataset” at the plugin's home page, the processing time in different computer systems range from 2 to 5 minutes (Table 1).

**Table:** 

**Computer setup**	**Time of execution with the Example Dataset**
11th Gen Intel(R) Core(TM) i7-1165G7 @ 2.80GHz - Solid State Drive - 16 GB RAM (Windows)	2 minutes 51 seconds
11th Gen Intel(R) Core(TM) i7-1165G7 @ 2.80GHz – Solid State Drive – 32 GB RAM (Windows)	2 minutes 22 seconds
Processor Intel(R) Core(TM) i7-7500U CPU @ 2.70GHz 2.90 GHz – Solid State Drive – 16 GB RAM (Windows)	4 minutes 49 seconds
Processor 2,3 GHz Intel Core i5 – 8 GB RAM (MAC)	3 minutes 24 seconds
Intel® Core™ i7-9700K × 8 – Solid State Drive - 64GB (Ubuntu)	1 minute 50 seconds

Table 1. Multi-Channel Nuclear Analysis plugin processing time using the Example Image Dataset available at the link provided in the Extended Data.

The Multi-Channel Nuclear Analysis tool addresses several key challenges in quantitative fluorescence microscopy:

1. Ease of Use: The graphical user interface eliminates the need for programming expertise during the data extraction from images, making advanced analysis accessible to a broader range of researchers.

2. Flexibility: Users can customize various parameters including channel settings, segmentation options, and measurement types.

3. Data Organization: The hierarchical organization of measurement data (individual channels → combined image tables → consolidated dataset) supports different analytical approaches and facilitates downstream analysis.

4. Batch Processing: The ability to process multiple images with consistent settings increases throughput and ensures reproducibility.

5. Complete Workflow Solution: The main analysis tool combined with the companion channel merging plugin provides a comprehensive solution for diverse microscopy data formats, accommodating both multi-channel files and systems that generate separate files for each channel.

The development of this tool followed the principles outlined by Pylva et al. (2025) for creating effective life science software, particularly focusing on user-centric design, clear documentation, and straightforward installation procedures. By incorporating these best practices, we aimed to bridge the gap between advanced image analysis capabilities and the needs of biological researchers.

The tool is particularly valuable for researchers studying nuclear morphology, chromatin organization, nuclear protein localization, and related phenomena. By combining accurate nuclear segmentation with multi-channel analysis, it enables quantitative assessment of relationships between nuclear features and other cellular components or molecular markers.

Potential limitations include the dependency on StarDist for segmentation, which may not be optimal for all nuclear morphologies, and the temporary compatibility limitation with Java 21-based FIJI versions as noted in the Implementation section. Users should ensure they are using the stable Java 8 version of FIJI until the underlying dependencies are updated. Future developments could include support for additional file formats, extended segmentation options, integration with machine learning approaches for feature selection and classification, and compatibility with newer Java versions as the dependent plugins evolve.

**Data availability**

The Multi-Channel Nuclear Analysis macro is freely available on GitHub via the link provided in the Extended Data. Plugin tutorials, example images and the Python script used for visualization are also provided.

## Methods

**Implementation**

Multi-Channel Nuclear Analysis was implemented as an ImageJ/FIJI macro using the built-in macro language. The tool utilizes the StarDist plugin for nuclear segmentation and core ImageJ functionality for image processing and measurements. The plugin is designed to analyze multi-channel fluorescence images in TIF and CZI format, though it can be adapted for other formats. This tool can be accessed via the link provided in the Extended Data. A detailed user guide, video tutorial, and example images and scripts for analyses are also provided.

**Compatibility Note**

The plugin requires the StarDist and CSBDeep plugins, which currently depend on TensorFlow functionality that is only compatible with Java 8. As of the publication date, this means the plugin works with the stable versions of FIJI (which run on Java 8) across all platforms (Windows, MacOS, and Linux), but not with the latest FIJI versions that use Java 21. Users can verify their FIJI's Java version by going to Help > About ImageJ and checking the Java version in the information displayed. If using the latest FIJI version, users should download the stable Java 8 version from the official FIJI download page (https://imagej.net/software/fiji/downloads). This compatibility limitation is temporary and will be resolved once the StarDist and TensorFlow plugins are updated to support Java 21.

**Companion Channel Merging Tool**

While the main analysis tool processes multi-channel images, we recognized that some microscopy systems (such as Evos microscopes from ThermoFisher Scientific) save each channel as a separate TIF file rather than a consolidated multi-channel file. To address this common workflow challenge, we developed a companion plugin called "Merge_multi_channel_tif.ijm" that prepares such data for analysis with the main tool. This tool can be accessed at via the link provided in the Extended Data..

This companion plugin identifies separate channel files with the same base name, but different channel suffixes, merges them into a single multi-channel TIF file, and preserves appropriate channel assignments and color look-up tables (LUTs). It features a similar user-friendly interface with configurable parameters for channel identification and merging options. Users can specify channel suffixes (e.g., "-DAPI", "-GFP", "-mCherry") and assign appropriate colors to each channel.

For this merging tool to work correctly, input files must follow a consistent naming convention, with each set of files sharing a common base name and having unique channel-specific suffixes at the end of the filename (e.g., "10X-Image1-DAPI.tif", "10X-Image1-GFP.tif"). The merged files are saved in a designated output directory, ready for processing with the main analysis tool.

**Workflow**

The analysis workflow for the Multi-Channel Nuclear Analysis plugin consists of four main steps ( Figure 1A ):

1. **Configuration** : A three-step GUI allows users to:

- Select the number of channels to analyze (1-4) and configure measurement parameters

- Define channel-specific settings including background subtraction value, maximum display value, color, and naming

- Select which channel to use for segmentation and which to include in the merged visualization.

2. **Nuclear Segmentation** : The selected segmentation channel is processed using StarDist's "Versatile (fluorescent nuclei)" model with configurable probability threshold, NMS threshold, and boundary exclusion parameters.

3. **Multi-Channel Analysis** : For each active channel, the macro:

- Applies background subtraction and display range settings

- Uses the segmented regions of interest (ROIs) to measure selected parameters

- Saves individual PNG channel images with appropriate display settings

4. **Data Organization** : The macro generates:

- Individual measurement tables for each channel

- Combined measurement tables for each image with appropriate header prefixes

- A consolidated table combining data from all processed images

**Data Outputs**

The tool generates several output files in an "analysis" subfolder:

- Individual channel images (PNG format)

- Segmentation visualization with ROIs overlaid

- Merged multi-channel visualization (optional)

- Individual channel measurement tables (CSV format)

- Combined measurement tables per image (CSV format)

- Consolidated measurement table for all images (CSV format)

- ROI sets (ZIP format)

- Analysis parameters record (TXT format)

**Data availability**

The Multi-Channel Nuclear Analysis macro is freely available on GitHub via the link provided in the Extended Data. Plugin tutorials, example images and the Python script used for visualization are also provided.

## Data Availability

Description: Source code for plugin, additional scripts, and Example Data Images. Resource Type: Software. DOI: https://doi.org/10.22002/6setz-n9180
